# Unilateral cerebellar hypoplasia

**DOI:** 10.4103/0971-3026.50838

**Published:** 2009-05

**Authors:** JD Vagh, Ajit Gadekar, Amit Agrawal, Kirti Deshmukh

**Affiliations:** Department of Pediatrics, Datta Meghe Institute of Medical Sciences, Sawangi, Meghe, Wardha, India; 1 Department of Surgery, Datta Meghe Institute of Medical Sciences, Sawangi, Meghe, Wardha, India

**Keywords:** Cerebellum, cerebellar hypoplasia, hypoplasia

## Abstract

Unilateral cerebellar hypoplasia is a relatively rare malformation. We report the case of a 7-year-old boy who presented with a history of a fall, which was followed by cerebellar signs. Imaging findings suggested a diagnosis of unilateral cerebellar hypoplasia. The child recovered with conservative management, probably because the cerebellar signs were due to the trauma and not the hypoplasia itself.

## Introduction

Unilateral cerebellar hypoplasia is a relatively rare malformation.[1–4] We report an unusual case of unilateral cerebellar hypoplasia that was detected following head injury in a previously asymptomatic child.

## Case Report

A 7-year-old male child presented with a history of fall from a height 8 days earlier. Following the fall, the child had a nasal bleed and multiple episodes of vomiting. He had difficulty in walking and his speech was slurred, with a nasal intonation.

His past history revealed that following delivery he had been admitted in the intensive care unit because of low birth weight and respiratory distress and had recovered.

On examination he had gait ataxia, hypotonia, and slurred speech. The plantar reflexes were extensor. The deep tendon reflexes were exaggerated on the right side.

**Figure 1 (A–C):**
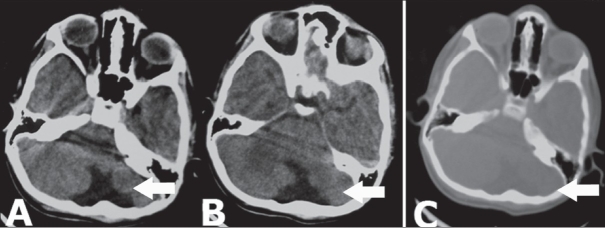
Axial CT scan images show hypoplasia of the left cerebellar hemisphere (arrows in A,B) with asymmetry of the posterior fossa (arrow in C)

**Figure 2 (A–D):**
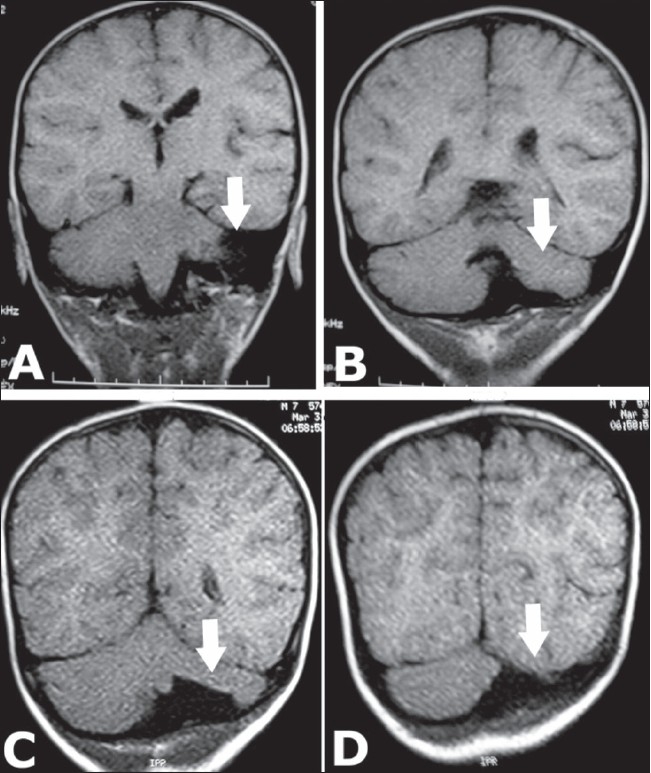
T1W coronal MRI images show left cerebellar hypoplasia (arrows)

A CT scan showed hypoplasia of the left cerebellar hemisphere, a large cisterna magna, and asymmetry of the posterior fossa: the left side being smaller than the right [Figure 1]. The smaller size of the left cerebellar hemisphere could be better appreciated on MRI [Figure 2]. Based on these imaging findings, a diagnosis of unilateral cerebellar hypoplasia was made. The child was managed conservatively for the head injury and he recovered.

## Discussion

On the basis of MRI findings, cerebellar malformations can be divided into those associated with hypoplasia and those with dysplasia; each type can show either focal or diffuse malformations. Focal cerebellar hypoplasia can be further subdivided into isolated vermis hypoplasia or hypoplasia of one cerebellar hemisphere.[3,4] Pathologic evidence of cerebellar injury due to birth asphyxia is well described and, because of its high metabolic activity, the vermis is the structure that is most commonly involved.[5] The clear demonstration of cerebellar hypoplasia, associated with hypoplasia or aplasia of the cerebellar or vertebral arteries, favors the concept of an intrauterine vascular etiology for cerebellar hypoplasia / aplasia.[2,6] Genetic mutations with somatic mosaicism may also have a role to play.[7] Also, as in our case, unilateral cerebellar hypoplasia may be an incidental finding in a patient with no previous evidence of neuromuscular or metabolic disease and no past history of trauma or anoxia.[1] However, cases with unilateral cerebellar hypoplasia can present with severe grand mal seizures, persistent headache,[8] or with psychomotor retardation without cerebellar symptomatology.[2]

CT scan shows posterior fossa asymmetry with underlying unilateral cerebellar hemisphere hypoplasia.[1,2] When available, MRI angiography may demonstrate the vascular anomalies in the cerebellar and / or vertebral arteries in most of the patients.[2] In the present case, the child's symptoms were primarily due to the head injury, which responded well to conservative management; the cerebellar hypoplasia was an incidental finding.
